# Cyclophilin A from Schistosoma japonicum promotes a Th2 response in mice

**DOI:** 10.1186/1756-3305-6-330

**Published:** 2013-11-17

**Authors:** Jinghui Li, Wenjia Zhuang, Li Cong, Wenjun Shi, Xingyan Cai, Fengjuan Huang, Yiteng Liao, Yiyang Liu, Jun Li, Chunxia Chen, Xiao-Ping Chen

**Affiliations:** 1 Department of Immunology, Tongji University School of Medicine, Shanghai 200092, China; 2 Department of Pathogen Biology, Tongji University School of Medicine, Shanghai 200092, China; 3 Shanghai Institute of Pharmaceutical Industry, Shanghai 200040, China

**Keywords:** Th2 response, *Schistosoma japonicum*, Cyclophilin A

## Abstract

**Background:**

Schistosomiasis is a chronic infection, where the host immune response to the parasite changes from a predominantly Th1 to Th2 phenotype, when parasite enters the egg stage, restraining the host inflammatory immune responses to achieve a longer survival in the host. On the other hand, the development of Th2 responses causes immunopathological changes such as liver fibrosis. Therefore identification of schistosome-derived Th2 inducing molecules is important in the understanding of pathogenesis of schistosomiasis. A cyclophilin A homologue of *Schistosoma japonicum* was reported to be an egg-stage specific antigen, but its immunogenicity and immunoregulatory activities remain unknown.

**Methods:**

We cloned and expressed the gene of cyclophilin A from *Schistosoma japonicum* (AY814078), named as SjCyP18 based on its molecular weight. The expression profiles in different stages of *S. japonicum* were examined by RT-PCR and immunofluorescence assay. The immunogenicity of SjCyP18 was measured by the presence of IgG in the sera from *S. japonicum* infected patients and animals, and the Th2-promting activities were examined by the subclass of immunoglobulins against SjCyP18 and by the IL-4 induction in T cells following footpad injection of SjCyP18.

**Results:**

The cloned SjCyP18 has 65% homology with human or mouse cyclophilin A at the amino acid level. In contrast to reports as an egg-stage specific antigen, the gene was found to be expressed in all stages of *S. japonicum*. IgG responses against SjCyP18 were found in some *S. japonicum* infected patients and were significantly induced when infection become patent and produce eggs in infected mice. Furthermore, the Th2-promoting subclass of IgG1 was the predominant isotype in *S. japonicum* infected mice. More importantly, footpad injection of SjCyP18 induced a greater production of IL-4 than that of IFN-γ by lymphocytes compared to responses from PBS injection controls.

**Conclusion:**

The cyclophilin A homologue found in *S. japonicum* is immunogenic and promotes Th2 responses *in vivo* which may contribute to the establishment of chronic infection by schistosomes.

## Background

Schistosomiasis is endemic throughout the tropics and subtropics. Infection by *Schistosoma japonicum* remains an important public health burden in South and East Asia despite the efforts of ongoing control programmes [1,2]. In contrast to many other infectious organisms, schistosome parasites are able to survive in the infected host for years, or even decades. This worm has developed effective strategies to express molecules homologous to those from the host (molecular mimicry) to facilitate evasion of host immune surveillance. Indeed, recent studies on the transcriptome information from *S. japonicum* indicated that hundreds of parasite genes share similarities with host homologues [3,4].

The other common mechanism used by this worm to evade host immune defense is to modulate inflammatory immune responses to facilitate the establishment of chronic infection in the host. The immunomodulatory cytokine IL-10, for example, can be induced by cells of both innate and adaptive immunity including regulatory T cells in response to egg- or worm-derived antigens [5]. Ablation of IL-10 resulted in exaggerated granulomatous disease and decreased host survival [6]. More importantly, the well-described IL-4-producing Th2 responses, induced when schistosome infection becomes patent and produces the egg-stage, contribute directly to the limitation of host pro-inflammatory reactions and the establishment of chronic infection. Examination of schistosome infection in mice defective for Th2 associated responses has shown that the inability to develop Th2 responses to restrain the initial pro-inflammatory response exacerbates acute schistosomiasis leading to earlier death [7,8]. Therefore, the Th2 response induced by schistosome infection is essential for the survival of both the host and the helminth worms with the cost of chronic schistisomiasis characterized by liver granulomatous inflammation and liver fibrosis.

It has been well established that egg antigens are primarily responsible for the Th2 induction [7]. The egg-derived IPSE/alpha-1 and omega-1, which are both glycoproteins secreted by the eggs have recently been shown to be involved in the initiation or potentiation of Th2 development in *S. mansoni* infection. IPSE/alpha-1 is able to trigger basophils to produce IL-4, which may contribute to Th2 polarization [9]. Omega-1, on the other hand, potently instructs DCs to prime highly Th2-polarized responses [10]. However, neither of these two molecules are fully responsible for the Th2 induction activities by soluble egg antigen (SEA) [10]. Therefore, more SEA-associated Th2-inducing molecules await characterization. In terms of *S. japonicum*, most of the identified molecules induce IFN-γ-dominant Th1 responses [11,12], except SjEsRRBL1, which is reported to elicit a mixed Th1/Th2 response [13]. The IPSE homologue cloned from *S. japonicum* failed to demonstrate stimulation of IL-4 production by basophils and no Omega-1 homologue was readily identified in *S. japonicum* (data not shown). Thus, the Th2-inducing molecules from *S. japonicum* are not well known.

In order to identify Th2-promoting molecules from *S. japonicum*, we focused on antigens reported to be highly or uniquely expressed in the eggs. The gene AY814078 was originally identified via high throughput sequencing as showing 65% homology with human cyclophilin A (HsCyPA)( AAH05982) [3] and reported to be egg-stage specific. Known as the target for the immunosuppressive drug cyclosporin A [14], cyclophilin A has been shown to have other effects. For example, cyclophilin A was reported to achieve anti-inflammatory effects both *in vivo* and *in vitro* via interacting with the membrane protein CD147 [15]. Furthermore, cyclosporin A demonstrated antimicrobial activity against a variety of eukaryotic pathogens including anti-schistosome effects [16]. The signature region making up the putative cyclosporin A-binding pocket is well conserved among cyclophilin A molecules from different organisms [17]. Although a number of cyclophilins have been uncovered in *S. mansoni* and *S. japonicum*[18-21], whether or not they are fully responsible for the anti-helminth activities by cyclosporin A remain unknown. Equally unknown are the immunoregulatory activities of these schistosome cyclophilins.

In this study, we cloned and expressed the gene AY814078, characterized its expression in different stages of worm development and analyzed its immunogenicity in infected mice and humans and its ability to induce Th2-polarized responses in mice. We found that cyclophilin A in *S. japonicum* (SjCyP18) is a homologue of human HsCyP18, which is highly expressed in adult worms, eggs and in soluble egg antigens. Furthermore, footpad injection of SjCyP18 induced IL-4 production by activated lymphocytes isolated from draining lymph nodes, corresponding to a robust Th2-promoted IgG1 subclass found in *S. japonicum* infected mice.

## Methods

### Animals and infection

6–8 weeks old female C57BL/6 mice were purchased from SLAC Lab Animals CO (Shanghai). All mice were maintained under specific pathogen-free conditions and fed with standard laboratory food and water. All procedures performed on animals within this study were conducted in accordance with and by approval of the Internal Review Board of Tongji University School of Medicine. Mice were challenged by skin-penetrating infection of 20 cercaria of *S. japonicum*, which were shed from infected *Oncomelania hupensis* snails provided by National Institute of Parasitic Diseases at Shanghai, China. The animal protocol was approved by the Committee on the Ethics of Animal Experiments of Tongji University (Permit Number: 2009–0022). The human study was approved by the Ethics Committee of Tongji University.

### Preparation of S. japonicum samples

The adult schistosomes were collected by perfusion from the portal vein of mice 42 days after infection. The soluble egg antigens (SEA) were made by the method developed by Brindley with modifications [22]. Livers from *S. japonicum* infected mice or livers from infected rabbits which were kindly provided by National Institute of Parasitic Diseases at Shanghai, China were chopped and homogenized in cold phosphate-buffered saline (PBS). 1 mg/ml collagenase IV, 10 μg penicillin and 10 μg streptomycin were added to the liver homogenate followed by incubation with shaking at 37°C overnight. The homogenate was centrifuged at 400 g for 5 min and the pellet resuspended in 3 ml of PBS and applied to the top of a Percoll column prepared by mixing 8 ml of Percoll with 32 ml of 0.25 M sucrose. The schistosome eggs were enriched in the pellet after centrifugation at 800 g for 10 min. The purified eggs were washed 3 times in PBS containing 1 mM EDTA and 1 mM EGTA at 30 g. Further purification of eggs was achieved by resuspension of the eggs in 0.5 ml of PBS and re-application on to a Percoll column prepared by mixing 2.5 ml of Percoll with 8.5 ml of 0.2 M sucrose and was pelleted and washed as before. Purified eggs were ultrasonicated for 10 s with an interval of 10s for 10 min in 5 ml PBS, and final soluble egg antigens (SEA) were obtained from the supernatants after being centrifuged at 400 g for 15 min and were filtered through 0.22 μm filters.

### Cloning, expression and purification of recombinant SjCyP18 and HsCyP18 and generation of anti- SjCyP18 mouse serum

Total RNA was isolated from eggs with TRIZOL reagent (Invitrogen) according to the manufacturer’s instructions. First strand cDNA was synthesized using reverse transcriptase AMV and oligo (dT) primer (Invitrogen) as described below. The primers were designed based on the posted sequence from AY814078 as sense primers 5′-GCC*GGATCC*TCAACGTTTCCCAGGGT-3′ containing BamHI and ansisense primers 5′-GTC*CTCGAG*TTAGCATTGCCCGCAATTAAC-3′ containing XhoI. The SjCyP18 gene was obtained by PCR reaction following the amplification conditions as below, and a recombinant pET28a-SjCyP18 vector was constructed after enzyme cutting and ligation. Secreted products in the supernatants were collected from BL21 (Incubation for 1.5 h at 37°C until the OD600 reached 0.6, IPTG induction at 0.5 mmol/L at 37°C for 4 h) and were purified with Ni-containing column (His60 Ni Superflow Resin, Takara) and concentrated by Ultracel PL-10 (Millipore). The polymyxin B-Agarose (Sigma) treatment was applied to remove endotoxin. Purified protein was run on SDS-PAGE and analyzed with either Coomassie blue or western blot.

Antisera to SjCyP18 were raised in mice by injection of mice with 50 μg of purified recombinant SjCyP18 protein subcutaneously in the presence of complete Freund’s adjuvant CFA (Sigma) in 100 μl. Boosting injection was administered by 100 μl incomplete Freund’s adjuvant IFA with 25 μg recombinant SjCyP18 protein at day 14 and day 28 respectively. The serum obtained at day 42 was used in the study for western blot or immunofluorescence staining analysis.

### Detection of SjCyP18 by RT-PCR

Total RNA from cercariae, adult male and female worms and eggs was isolated by TRIzol (Invitrogen) according to the manufacturer’s instructions. 1 μg of RNA was reverse transcribed into cDNA with oligo dT primers in 20 μl containing 5 × RT buffer (4 μl),10 mM dNTP (1 μl), 10 μM Oliga dT (0.5 μl), RNase inhibitor (40 units) and AMV (5 units) at 42°C for 1 h. To exclude the possibility of genomic DNA contamination in RNA preparations, a parallel reaction without reverse transcriptase was performed. 2 μl cDNA or 2 μl RNA was used for PCR which was performed in a volume of 20 μl containing 2 × EasyTaq® PCR SuperMix (10 μl,TransGen Biotech), 20 μM primer (0.5 μl for each) with reaction as 94°C 5 min followed by 94°C 30 sec, 55°C 30 sec and 72°C 1 min for 30 cycles with final extension of 72°C for 5 min. The sequences of primers for PCR were the same as those for cloning primers.

### Detection of SjCyP18 by immunofluorescence staining

Polyclonal antibodies for SjCyP18 were raised in-house and used in diluted mouse serum (1:50 in TBST). Mouse antiserum against *S. japonicum* tetraspanins anti-TSP2 (1:50 dilution, provided by Institute of Infectious Disease and Vaccine Development, Tongji University) [23] was used as a positive control for immunostaining. The secondary antibody used was AlexaFluor 488-conjugated goat anti-mouse IgG (1:250, Invitrogen). The eggs, adult male and female worms and cercariae of *S. japonicum* were fixed in 10% buffered formalin for 10 min. The fixed organisms were frozen in OCT medium (Tissue-Tek) and stored at −80°C first and were then sectioned and mounted on glass slides coated with gelatin. Each section was cut to 10 μm thickness with Cryostat(Microm HM525) at −20°C. After being blocked with 10% bovine serum-containing medium for 1 h at room temperature, slides were dried and then washed three times for 2 min interval with PBST (PBS, pH 7.4 with 0.1% Tween-20). Slides were incubated with primary antibodies overnight at 4°C in PBS. After being washed in PBS, slides were incubated with secondary antibodies for 10 min at room temperature in the dark. The slides were mounted with Antifade Mounting Medium (Beyotime Institute of Biotechnology) after final wash with PBS. In addition to being stained by anti-SjCyP18, sections were also stained by either anti-SjTSP2-serum or normal mouse serum (5 normal sera were pooled) for positive and negative controls, respectively. Images were collected on an inverted fluorescence microscope. Staining was also carried out on fixed, whole-mount cercariae.

### Measurement of antibodies against SjCyP18 or against SEA in patients and infected mice by ELISA

Mouse sera were collected six weeks or at indicated infected time points after *S. japonicum* infection. ELISA was performed to determine antigen-specific total IgG, IgG1 and IgG2c. The 96-well microtiter plates were coated with either purified SjCyP18 at 5 μg/ml or SEA at 10 μg/ml in 50 mM carbonate buffer (pH9.6) overnight at 4°C, washed five times with PBST and blocked with 5% nonfat dry milk in PBST at room temperature for 1 h. After being washed five times, the wells were incubated with infected mouse serum at 1:200 dilutions at 37°C for 2 h. The plates were washed with PBST three times, followed by adding peroxidase-conjugated anti-mouse IgG at 1:2000 or anti-mouse IgG1 or anti-mouse IgG2c at 1:7500 (Santa Cruz) at 50 μl/well. After 2 h incubation at room temperature and wash, the final color development was achieved by adding peroxidase substrate ABTS (2,2′-Azino-bis(3-Ethylbenzthiazoline-6-Sulfonic Acid) (Sigma) to each well at 100 μl/well and absorbance was measured at 405 nm.

A similar method was used to measure the anti-SjCyP18 IgG present in sera from 5 normal human subjects and 10 patients who were positive for eggs in the feces by the Kato-Katz test. The sera were provided by Institute of Infectious Disease and Vaccine Development, Tongji University and were used at a dilution of 1:400. The detection was made by peroxidase-conjugated anti-human IgG (Promega) diluted at 1:2500.

### Induction of Th2 response in vivo

Mice were immunized subcutaneously in the hind footpad with 20 μg SEA or 15 μg SjCyP18 in a volume of 20 μl in PBS and the draining popliteal lymph nodes were removed aseptically from 4d immunized or naive mice. Lymphocyte suspensions were produced by passing through a 200 μm mesh and were stimulated by anti-CD3 and anti-CD28 antibodies (eBioscience) for 48 hours and the concentration of IL-4 and IFN-γ in the supernatants were analyzed as described [24].

### Statistical analysis

Unpaired Student’s t test and one-way or two-way ANOVA were used to determine the statistical significance of the differences among groups. Each individual experiment was conducted with at least 3 mice and each experiment was repeated at least twice.

## Results

### Cloning, expression and purification of SjCyP18

Belonging to the immunophilin family, cyclophilins are known to have important intracellular regulatory activities in T-cell activation. Intrigued by its high homology with HsCyP18 and the reported egg-stage-specific expression of AY814078 from *S. japonicum*[3], we cloned this gene from egg cDNA. The successfully cloned full-length cDNA contained 495 bp encoding an ORF of 164 amino acids with a theoretical molecular mass of 18 kDa, thus named as SjCyP18. Amino acid sequence alignment analysis showed that SjCyP18 is 66% identical to the human CyP18 (GenBank:AAH05982) or mouse CyP18 (GenBank:P17742) and 62% identical to *S. mansoni*-derived cyclophilin A SmCyP18 (GenBank:AAC47317) [19]. Comparing to a previously identified *S. japonicum*-derived cyclophilin A (GenBank:AAA29863) [18], 60% similarity was observed. Furthermore, a newly described *S. japonicum*-derived protein whose gene sequence is 99% identical to our AY814078 gene, was also characterized as *S. japonicum* cyclopilin A (GenBank: FN323717) [25].

To further study the function of SjCyP18, the prokaryotic expression vector pET28a-SjCyP18 was constructed and His-SjCyP18 fusion protein was abundantly induced and purified. The successful purification of SjCyP18 was verified by western blot either by anti-His antibodies or by polyclonal antibodies generated against SjCyP18 (data not shown).

### Analysis of expression of SjCyP18 on schistosomes

**Figure 1:**
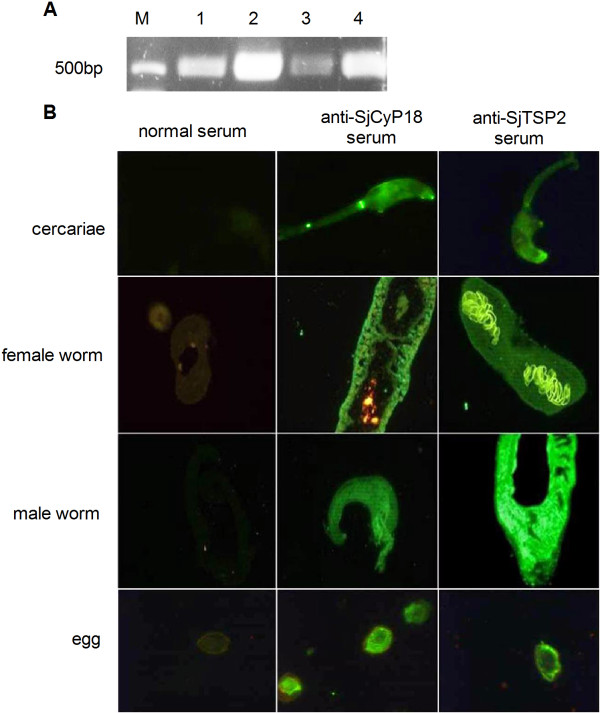
**Expression of SjCyP18 in different stages of *S. japonicum*. A** RT-PCR analysis of cDNAs from different stages of *S. japonicum* including cercariae (lane 1), male adult (lane 2), female adult (lane 3) and egg (lane 4). **B** Immunofluorescence staining of SjCyP18 in cercariae, female adult worms, male adult worms and eggs of *S. japonicum*. Parasite sections were reacted with anti-serum produced in mice against recombinant SjCyP18 and was then detected with Alexa Fluor 488 conjugated goat anti-mouse IgG. Preimmune normal serum and anti-TSP2-serum were used as negative and positive controls respectively.

**Figure 2:**
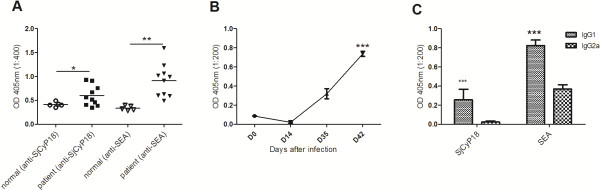
**Analysis of anti-SjCyP18 IgG in *S. japonicum *infected patients and anti-SjCyP18 IgG and its subclasses in infected mice. A** Sera from 10 *S. japonicum* infected patients who were positive by Kato-Katz test and from 5 normal subjects were measured for anti-SjCyP18 or anti-SEA IgG by ELISA. The significance test was analyzed by unpaired t-test with * as *p* < 0.05 and ** as *p* < 0.01. **B** The time course of anti-SjCyP18 IgG in *S. japonicum* infected mice, showing the mean ± SEM from 4 mice, representative of two separate experiments. One-way ANOVA was used to test the significance on the levels of day 42 with all the other time points with *** as *p* < 0.001. **C** Mouse sera from 42d *Sj* infected mice were measured for anti-SjCyP18 or anti-SEA IgG1 or IgG2c by ELISA as described in the methods. The results shown on IgG subclasses were the mean value of 4 mice and a representative of 5 separately performed experiments. Two-way ANOVA was used to test the significances of differences with *** as *p* < 0.001.

Unlike the reported result that SjCyP18 expression is egg-stage specific [3], our RT-PCR results demonstrated that this gene was expressed in all schistosome life cycle stages examined (cercariae, adults and eggs) (Figure 1A). We then applied immunofluorescence staining to observe the expression and localization of SjCyP18 in schistosomes in different life stages with mouse antiserum prepared against recombinant SjCyP18. The results showed that immunostaining were found in cercariae, female and male worms and eggs. The positive staining disappeared after preincubation of antiserum with recombinant SjCyP18 indicating that the positive immunostaining signals were specific (data not shown). Similar to the outer tegument staining pattern of TSP2 on adult *S.masoni*[26] and *S.japonicum*[23], the positive staining for SjCyP18 was also found at the surfaces of both female and male adult worms, except that the reproductive tissues in female worms was only positive for anti-TSP2 (Figure 2B).

### Anti-SjCyP18 IgG level is increased in S. japonicum infected patients

Due to similar surface expression pattern between SjCyP18 and TSP2 which indicates that SjCyP18 may also function as an immunogen, such as TSP2 [26], specific anti-SjCyP18 IgG was measured in serum samples from *S. japonicum* infected patients who were all positive for eggs in the feces by the Kato-Katz test. Similar to the increased levels of anti-SEA IgG in patients, the average level of anti-SjCyP18 IgG was also significantly higher in patients than that of normal sera (Figure 2A), suggesting that SjCyP18 is immunogenic during *S. japonicum* infection.

### Th2-associated IgG1 is predominantly induced in S. japonicum infected mice

When *S. japonicum* infected mice sera were examined, the levels of anti-SjCyP18 IgG were increased significantly between days 35 to 42 post-infection compared to those at 14 days after infection and in uninfected mice (Figure 2B). Furthermore, we tested the subclasses of the SjCyP18-specific IgG and found that Th2-associated IgG1 levels were higher than Th1-associated IgG2c levels, corresponding to the dominant IgG1 subclass found in infected mice when SEA was analysed (Figure 2C). These may indicate that SjCyP18 could contribute to induce a Th2-dominated immune response during post patent *S. japonicum* infection.

### Induction of Th2 response by SjCyP18 in vivo

**Figure 3:**
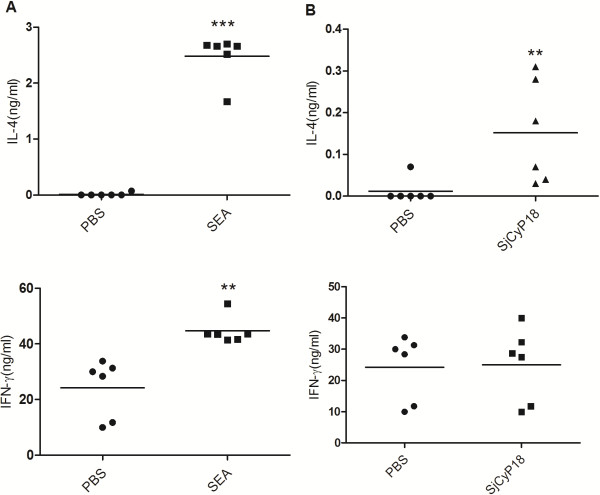
**Induction of Th2 response by footpad injection of SjCyP18.** Draining popliteal lymph node cells were isolated 4 days after SEA **(A)** or SjCyP18 **(B)** footpad injection. The IL-4 and IFN-γ concentration in the supernatants from lymphocytes reactivated with anti-CD3 and anti-CD28 antibodies were measured by sandwich ELISA as described in Methods. Results shown were representative results of five different experiments. Student’s t test was used to analyze the significance with ** as *p* < 0.01, *** as *p* < 0.001.

Since SjCyP18 appears to preferentially induce an IgG1-dominant response, its capacity to promote IL-4-production was then examined. When SEA was injected via footpad, lymphocytes from popliteal draining lymph nodes were found to produce large quantities of IL-4 following restimulation of lymphocytes with anti-CD3 and anti-CD28 for 48 hours *in vitro*, although IFN-γ production was also induced compared to PBS controls. When SjCyP18 was injected, IL-4 production was also significantly induced comparing to PBS control although at much lower levels than that induced by SEA injection. However, no enhanced induction of IFN-γ was observed comparing to PBS treatment (Figure 3). Therefore, SjCyP18 was able to promote a Th2 response *in vivo*.

## Discussion

Multiple cyclophilin isoforms have been reported in mammalian cells including HsCyPA also called HsCyP18, HsCyP40, HsCyPB, HsCyPC, HsCyPD, HsCyPE, HsCyPNK and others. HsCyPA and HsCyP40 are abundantly expressed in the cytosol in various types of cells; others may possess signal sequences that target them to specific organelles like ER and mitochondria [27]. As a prototypical minimal cyclophilin, which contains just the peptidyl-prolyl cis-trans isomerase (PPIase) domain and is the target for immunosuppressant drug cyclosporin A, CyP18 have also been described in prokaryocytes and eukaryotic pathogens like schistosomes and *Plasmodium falciparum*[28]. In this study, we reported a HsCyP18 homologue in *Schistosoma japonicum* and it is found to be expressed at all stages of life cycles in schistosomes and abundantly present in soluble egg antigens.

The activities of the widely expressed CyPA in mammalian and nonmamalian cells have not been definitely clarified except for its well-known role as a receptor for the immunosuppressive drug. A recent study on *Leishmania donovani* has suggested that CyPA played essential roles in proliferation and survival of the organism, which is independent of its PPIase activity [29]*.* On the other hand, several studies reported that CyPA can be secreted from the cells following inflammatory stimulation or oxygen stress [30]. Secreted CyPA can in turn enhance the adhesion activities, ROS production and proinflammatory activities on endothelial cells which is probably involved in the pathogenesis of arthrosclerosis [31,32]. Furthermore, CyPA possesses chemoattractant activities on neutrophils, eosinophils, monocytes and T cells [33], relating to the development of rheumatoid arthritis [34,35]. The recombinant SjCyP18 in this study failed to display chemotactic activities on human monocytes (data not shown).

Our studies, however, did demonstrate that the schistosome-derived CyPA played a role in the interplay between worms and host. It was an immunogen that was able to induce IgG production especially IgG1 in infected mice, corresponding to its capacity to promote a Th2 response when injected *in vivo*. Th2 responses are known to be a critical response for the helminth to subvert the host immune response create a co-existing state [36]. The responsible molecules present in schistosomes especially in SEA for the induction of Th2 response are not completely elucidated. In *S. mansoni* infection, omega-1 was largely responsible for the Th2-polarization by SEA *in vitro*, but *in vivo* experiments demonstrated that its role was limited [10]. To date, there is no omega-1 homologue reported in *S. japonicum* and sequence alignment analysis performed by us failed to find any significant homologue molecule in *S. japonicum* either (data not shown). Therefore, SjCyP18 is the first molecule reported from *S. japonicum,* which was able to induce a mild Th2 response *in vivo* independently. Moreover, we have reason to predict that there should exist other potent molecules in the SEA from *S. japonicum*, since the IL-4-promoting activities induced by SjCyP18 was much weaker than induced by SEA.

The potential of SjCyP18 as a vaccine candidate was also examined when the molecule was injected with or without CFA prior to *S. japonicum* infection. After three immunizations of SjCyP18, significant amount of anti-SjCyP18 IgG was induced (data now shown). Although a recent study declared that a *S. japonicum* cyclophilin A homologue protein could reduce the worm burden and liver egg burden in *S. japonicum* challenged mice [25], no significant protective effect was observed in our study (data not shown). The lack of protective effects by SjCyP18 immunization may be associated with the Th2-inducing ability of this molecule. Usually a Th1-dominant response is associated with protective effects in the *S. japonicum* infection model [37].

## Conclusions

In summary, we cloned and expressed the gene of *S. japonicum*-derived cyclophilin A, SjCyP18, which has about 66% homology with human CyP18 at the amino acid level. Furthermore, SjCyP18 was expressed by all stages of *S. japonicum* and was localized to the outer tegument of adult worms. IgGs against SjCyP18 were found in chronically infected patients as well as in mice during post-patent infections. *In vivo* injection of SjCyP18 facilitated an IL-4-producing Th2 response, corresponding to the dominant IgG1 response seen in infected mice. Altogether, our study demonstrates another important role played by a *S. japonicum*-derived cyclophilin in the interplay between host and pathogens.

## Abbreviations

SEA: Soluble egg antigen; CyPA: Cyclophilin A; HsCyPA: Human cyclophilin A; Sj: *Schistosoma japonicum.*

## Competing interests

The authors declare that they have no competing interests.

## Authors’ contributions

JL, WZ, WS and XPC participated in the design of the study. JL, XC and WS did the experiments on cloning, expression and serum response on SjCyP18. WZ and LC did the Th2 induction experiments. YL and YL cloned and expressed the HsCyP18. JL, WZ and XPC wrote the manuscript. All authors read and approved the final manuscript.
